# Predation Pressure on Sentinel Insect Prey along a Riverside Urbanization Gradient in Hungary

**DOI:** 10.3390/insects11020097

**Published:** 2020-02-01

**Authors:** Csaba Béla Eötvös, Gábor L. Lövei, Tibor Magura

**Affiliations:** 1 Department of Ecology, University of Debrecen, H-4032 Debrecen, Hungary; maguratibor@gmail.com; 2 Department of Forest Protection, NARIC Forest Research Institute, H-3232 Mátrafüred, Hungary; 3 Department of Agroecology, Aarhus University, Flakkebjerg Research Centre, DK-4200 Slagelse, Denmark; gabor.lovei@agro.au.dk

**Keywords:** urban, rural, ecological function, predation, predation paradox, sentinel prey, caterpillar, arthropod, mammal, bird

## Abstract

Urbanization is one of the most important global trends which causes habitat reduction and alteration which are, in turn, the main reasons for the well-documented reduction in structural and functional diversity in urbanized environments. In contrast, effects on ecological mechanisms are less known. Predation is one of the most important ecological functions because of its community-structuring effects. We studied six forest habitats along a riverside urbanization gradient in Szeged, a major city in southern Hungary, crossed by the river Tisza, to describe how extreme events (e.g., floods) as primary selective pressure act on adaptation in riparian habitats. We found a generally decreasing predation pressure from rural to urban habitats as predicted by the increasing disturbance hypothesis (higher predator abundances in rural than in urban habitats). The only predators that reacted differently to urbanization were ground active arthropods, where results conformed to the prediction of the intermediate disturbance hypothesis (higher abundance in moderately disturbed suburban habitats). We did not find any evidence that communities exposed to extreme flood events were preadapted to the effects of urbanization. The probable reason is that changes accompanied by urbanization are much faster than natural landscape change, so the communities cannot adapt to them.

## 1. Introduction

Urbanization is as old as the first cities which appeared between 5100 BC and 2900 BC in the Fertile Crescent [1]. Today, urbanization is one of the most important processes shaping our environment, with fewer people living in rural than urban areas globally [2]. By 2050, the global rural population is expected to be ca. 3.1 billion, slightly less than today, while the urban populations are projected to reach 6.7 billion [2]. Globally, urbanization has several similar elements. From rural areas to urban centres, the original habitat matrix becomes smaller and more fragmented, road densities increase, along with the area covered by artificial surfaces, with air and soil pollution often showing the same trend [3,4]. Other changes include more human disturbance, increased noise level, and changes in temperature and precipitation patterns [5].

The biodiversity of the area affected by urbanization changes significantly. The urban matrix is rarely suitable for most of the original inhabitants of the rural habitat [6,7], so there is a reorganization of biodiversity [8,9]. Besides the changes in habitat amount, distribution and quality, new sources of mortality—including novel xenobiotics, hunting, collision with structures or vehicles, electrocution, and predation in the urban environment—can decrease survival rates [10]. Hence, several animal and plant species present in the original rural habitat decrease in density or disappear altogether from urban habitats [11] and are often replaced by non-native species [12,13]. Effects of urbanization can also cause several physiological and behavioural changes in body size [14], body size distribution [15], fluctuating asymmetry [16], migratory behaviour [17], as well as lower reproduction [18] and survival rates [19]. Habitat specialists often disappear from urbanized areas [20,21,22] and consequently, functional diversity can decrease [23]. However, urbanization does not necessarily result in losses in taxonomic diversity or species richness [24]. More importantly, urbanization can change ecological functioning such as biogeochemical cycles [25] and trophic interactions (pollination [26], parasitism [27], predation [28,29]).

Predation, due to its community structuring effects, is one of the most important ecological processes [30]. Predator assemblages of urban habitats are different from those in rural ones [31,32]. Many predators avoid urban habitats, at least during daytime [33]. Populations of synanthropic predators can reach higher abundance in urban environments [31]. Local prey distribution can be changed by prey aggregating near light sources [34] or bird feeders [35]. Predator populations react to this [36], causing increased local predation rates [37]. Reaction to altered predation risk can lead to changes in behaviour [38], demographics, and interspecies interactions [39]. Current evidence indicates that predation pressure is lower in urban than rural areas, but this is mostly based on data on vertebrate predators; there is very little quantitative information about invertebrate predation [28].

Quantification of predation on invertebrate prey is difficult, because the attacks are mostly cryptic and evidence is difficult to obtain. Visual or video surveillance is complicated and expensive, and the activity of the predator may be affected by the observer or the equipment [40]. Gut content analysis or prey labelling produces results of varying resolution and secondary predation and the spread of the label can be related to non-predatory events [41]. Sentinel prey is one widely used method to measure predation intensity [42]. The prey can be immobile stages of arthropods such as eggs [43], pupae [44], or immobilized insects (e.g., aphids glued on self-adhesive paper [45]). The use of real prey is an advantage, but the identity of the predator usually remains unknown [42]. In contrast, artificial prey are not removed and the attack marks left by predators allow identification [46]. This method is suitable to compare predation pressure in various habitats [46].

We used the sentinel prey method to characterize predation on artificial caterpillars along an urbanization gradient in Szeged, Hungary. The specialty of this location is that this city—similarly to all the 50 largest built-up urban areas [47] except Mexico City—is built next to water. In spite of this feature, studies on ecological mechanisms in cities do not commonly consider this important factor. According to the natural flow-regime paradigm [48,49], extreme events (e.g., flood) exert a strong environmental filter in riparian habitats. With this in mind, we tested the following hypotheses:

**H1.**  *The drastic, frequent floods constitute such a powerful environmental filter that other effects of urbanization are overwritten. Thus, there would be no difference in predation pressure along the gradient because floods equally affect all stages of the urbanization gradient.*

**H2.**  *Predation rates are lower in more urbanized habitats than in the rural ones. According to the increasing disturbance hypothesis [50], predator abundance decreases with advancing urbanization, and thus predation pressure would also decrease.*

**H3.**  *Bird predation will show a peak during breeding time. According to the match/mismatch hypothesis [51] the reproductive success of birds is maximized when they synchronize their reproduction with the peak of the food supply [52], around mid-May in Hungary [53].*

**H4.**  *Predation by small mammals will peak at the end of the growing season. The reproductive cycle of small mammals such as the wood mouse, Apodemus sylvaticus (L., 1758), in Europe shows its minimum in winter and its maximum in summer, producing the lowest population abundance in spring and summer, while the highest population abundances are in autumn and winter [54]. Consequently, we can expect the highest predation activity by small mammals during the autumn study period.*

We found the highest predation rates in rural habitats by all predator groups except arthropods at ground level, refuting H1 while partially supporting H2: the effects of urbanization can overwrite the effects of regular floods. Predation activity by birds and arthropods was the highest in spring and summer (H3 supported), while the highest peak of predatory activity by small mammals was recorded in summer (H4 not supported).

## 2. Materials and Methods

**Figure 1:**
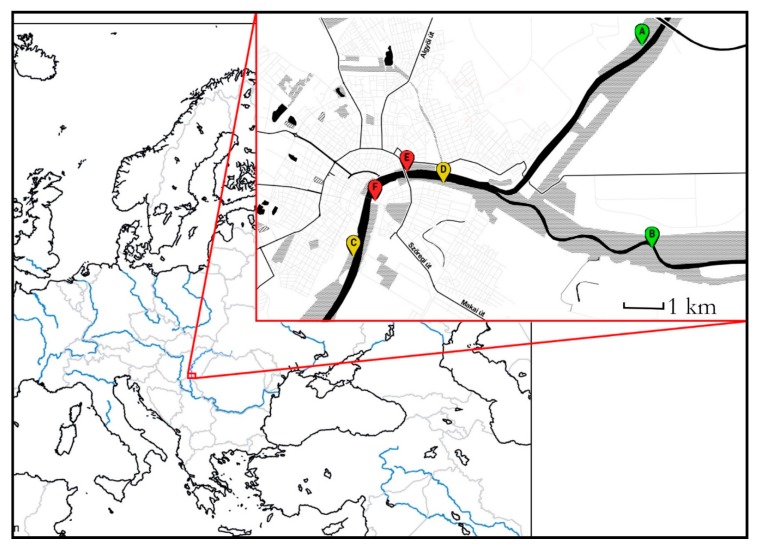
The map of the study sites. A and B rural, C and D suburban, and E and F urban sites.

Our study site was in and around the city of Szeged (46°15′ N; 20°8′ E), 170 km southeast of Budapest (Hungary). This city lies on both sides of the lower reaches of the Tisza River. Upstream but within the city limits, it also receives the Maros River: both of them collect water from the Carpathians, and regularly flood their forested floodbeds (although no flood was registered during the period of study) (Table A1). The floodbed was transformed during the end of the 19th century so, that the drainage of the water is optimal and the flood effect reducing infrastructure, built in 1973, is far enough upstream (150 km) from the city to assume equal flood intensity across the urban gradient [55]. The riverside forests are nearly continuous on the left bank, and more fragmented on the right one, allowing us to choose an urbanization gradient fulfilling the Globenet protocol conditions [56] (Figure 1).

Vegetation originally was a primary forest of white willow (*Salix alba*) and white poplar (*Populus alba*). In the last decades, two invasive tree species [57], green ash (*Fraxinus pennsylvanica*) and box elder (*Acer negundo*) encroached on the area [55]. The undergrowth was dominated by invasive species, with a few natives (Table 1).

**Figure 2:**
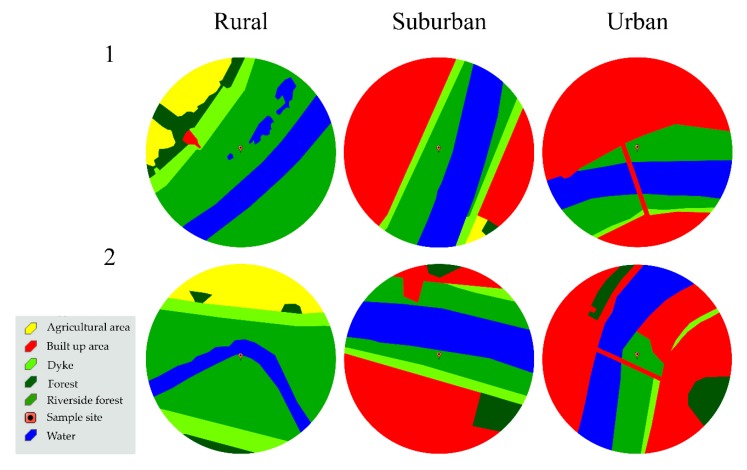
Habitat types within a 500-m radius of the study plots.

Data were collected during the growing seasons (April–October) in 2014–2016. Selected locations on both banks included rural, suburban, and urban areas, with an increase in the built-up area, increasing intensity of forest management, as well as visitation rates by city residents from rural to urban habitats (Figure 2). The average built up area (within a 500 m radius of a study site) was 0.3% in rural, 41.3% in suburban, and 54.8% in urban sites (Figure 2). In rural areas, cultivated fields dominated outside the dykes (12.5%) (Figure 2). In the riverside forest, the undergrowth was not managed in the rural sites, while in the urban sites the undergrowth was cut twice yearly. In the suburban areas, the last such operation was 2 years before the start of the study.

**Figure 3:**
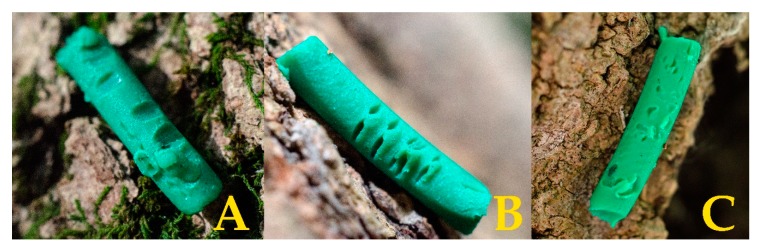
Characteristic marks left by different predator groups on dummy caterpillars. **A**—small mammals, **B**—arthropods, **C**—birds.

Along the two urbanization gradients, the average distance between sites was 5 km (range: 1–10 km). In each site, there were four patches with a minimum distance of 10 m between them. Within each patch, 12 trees were selected pseudo randomly by considering the species and trunk diameter. The average distance of selected trees within patches were 4.4 m (range: 0.5–23 m). On each of these, one dummy caterpillar was placed on the trunk and one at a random distance (range: 0–4 m) and direction from the base of the tree, on the ground. The dummy caterpillars (20 mm long, 3 mm thick) were made of light green plasticine (Smeedi plus, V. nr. 776609, Vilborg, Denmark), using a modified garlic press [46]. The colour, shape, and size imitated a general caterpillar prey [42]. The artificial prey was fixed to the bark of the trees or on a suitable surface on the ground with superglue (Pentack Super Glue, Pentacolor, Budapest, Hungary), and exposed for 24 h, then checked for attack marks using a handheld magnifying glass (10 ×). In case of doubt, the caterpillar was photographed and inspected on computer. Predators were identified by their characteristic marks left on the artificial prey (Figure 3). Attacks by different predators were considered independent events, but multiple marks by the same type of predator were classified as single attack. Overall, 12,672 caterpillars were exposed during the three years; 448 of them were not recovered.

For temporal analysis, May and June were considered spring, summer during July and August and autumn from September to the end of October.

### Data Analysis

We analysed data on ground-placed vs. trunk-placed caterpillars separately.

Before the start of the analysis, spatial autocorrelation was checked to decide whether the individual caterpillars could be considered independent, using Moran’s I [58].

We used generalized linear mixed model (GLMM) [59,60] with urbanization stage and season as fixed effects, and study site as random factor. We found no significant differences among predation levels between the two gradients and the study years, so these factors were omitted.

Lognormal distribution fitted to our data best on the quantile to quantile plot, allowing us to use the penalized quasi-likelihood (PQL) method. PQL is a flexible technique that can deal with non-normal data, unbalanced design, and crossed random effects [61]. It effectively treats the random effects as ‘fixed’ and estimates them in a similar manner to other fixed effects as in a generalized linear model (GLM). Under a growing number of clusters, PQL estimates remain estimation consistent [62]. For multiple comparison of means we used Tukey test.

All calculation were made in R (version 3.5.1) [63]. Packages ncf [64] and lme4 [65] were used for autocorrelation methods. For GLMM calculations, we used car [66], MASS [67], and nlme [68].

Some of the marks were not related to predator attack, and in wet weather, snails occasionally left characteristic trails. In such case, the caterpillar was considered missing. A difference was considered significant at *p* < 0.05.

## 3. Results

There were 1780 (14.6%) attacks on sentinel prey. At ground level from the 6336 preys, 974 (16%) were attacked. On the soil surface small mammals were the most active predators (10.4%), followed by birds (3.4%), and arthropods (2.6%) (Table 2). From the trunk-placed 6336 caterpillars, 836 (13.2%) were attacked. On tree trunks, arthropods were the most active predators (8.8%), followed by small mammals (3.0%) and birds (1.2%) (Table 2). We were not able to identify predation marks on 448 (3.5%) dummy caterpillars because they were either missing or melted (Table 2).

### 3.1. Predation Levels along the Urbanization Gradient

#### 3.1.1. Overall Predation on Dummy Caterpillars

**Figure 4:**
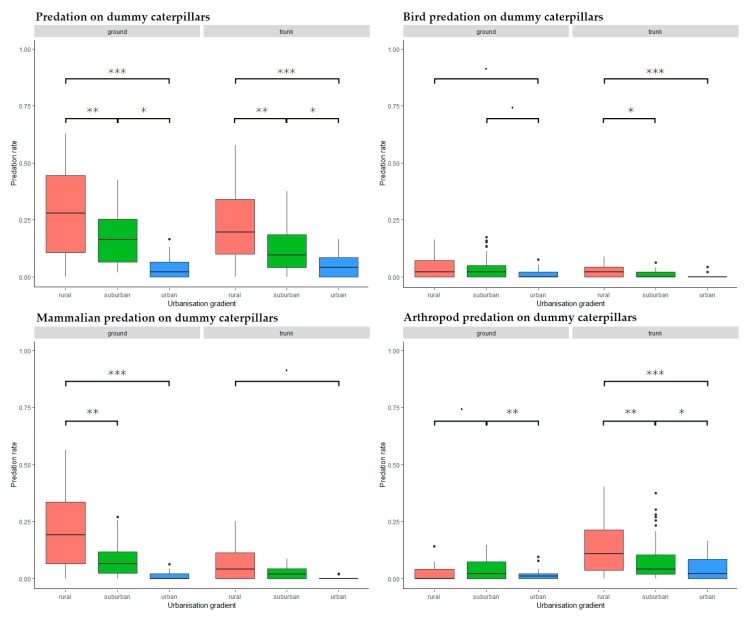
Predation activity along the urbanization gradient on different placement and by different predator groups. Significance codes: ***: *p* < 0.001, **: *p* < 0.01, *: *p* < 0.05, **˙**: *p* < 0.1.

We found a significant, decreasing trend in attack frequency from rural to urban habitats both on trunk (rural–suburban: Estimate = −0.087, SD = 0.025, z = −3.555, *p* = 0.001, rural–urban: Estimate = 0.152, SD = 0.025, z = 6.098, *p* < 0.001, suburban–urban: Estimate = 0.065 SD = 0.025, z = 2.553, *p* = 0.029) and on ground level (rural–suburban: Estimate = −0.096, SD = 0.036, z = −2.700, *p* = 0.019, rural–urban: Estimate = 0.213, SD = 0.036, z = 5.921, *p* < 0.001, suburban–urban: Estimate = 0.118, SD = 0.036, z = 3.231, *p* = 0.004) (Figure 4).

#### 3.1.2. Bird Predation on Dummy Caterpillars

There was a generally decreasing trend along the urbanization gradient (Figure 4). We found no significant differences in attack rates at ground level (rural–suburban: Estimate = −0.003, SD = 0.013, z = −0.241, *p* = 0.090, rural–urban: Estimate = 0.031, SD = 0.013, z = 2.342, *p* = 0.050, suburban–urban: Estimate = 0.028, SD = 0.013, z = 2.101, *p* = 0.969), but trunk-placed caterpillars in the rural habitat suffered significantly higher attack rates than those in the suburban or urban habitats (rural–suburban: Estimate = −0.014, SD = 0.006, z = −2.377, *p* = 0.046, rural–urban: Estimate = 0.022, SD = 0.006, z = 3.648, *p* < 0.001, suburban–urban: Estimate = 0.008, SD = 0.006, z = 1.271, *p* = 0.412) (Figure 4).

#### 3.1.3. Mammalian Predation on Dummy Caterpillars

Similarly to birds, mammals showed a decreasing trend of predation activity along the urbanization gradient (Figure 4). However, mammal predation at ground level in rural habitats was significantly higher than in suburban or urban ones (rural–suburban: Estimate = −0.116, SD = 0.038, z = −3.067, *p* = 0.006, rural–urban: Estimate = 0.186, SD = 0.038, z = 4.906, *p* < 0.001, suburban–urban: Estimate = 0.071, SD = 0.038, z = 1.843, *p* = 0.156) (Figure 4). No significant difference was found on caterpillars placed on tree trunks (rural–suburban: Estimate = −0.035, SD = 0.028, z = −1.253, *p* = 0.422, rural–urban: Estimate = 0.059, SD = 0.028, z = 2.081, *p* = 0.094, suburban–urban: Estimate = 0.023, SD = 0.028, z = 0.828, *p* = 0.686) (Figure 4).

#### 3.1.4. Arthropod Predation on Dummy Caterpillars

Arthropod attacks showed a decreasing trend along the urbanization gradient from rural to urban habitats on tree trunks but not on ground level (Figure 4). At ground level, suburban predation activity was significantly higher than in urban habitats, with a trend of higher activity in suburban than rural habitats (rural–suburban: Estimate = 0.019, SD = 0.009, z = 2.205, *p* = 0.070, rural–urban: Estimate = 0.007, SD = 0.009, z = 0.830, *p* = 0.685, suburban–urban: Estimate = 0.026, SD = 0.009, z = 3.035, *p* = 0.007) (Figure 4). On trunk placed prey, we found significantly decreasing predation levels from the rural to the urban habitats (rural–suburban: Estimate = −0.048, SD = 0.015, z = −3.260, *p* = 0.003, rural–urban: Estimate = 0.085, SD = 0.015, z = 5.628, *p* < 0.001, suburban–urban: Estimate = 0.037, SD = 0.015, z = 2.377, *p* = 0.046) (Figure 4).

### 3.2. Seasonal Trends

#### 3.2.1. Overall Predation on Dummy Caterpillars

**Figure 5:**
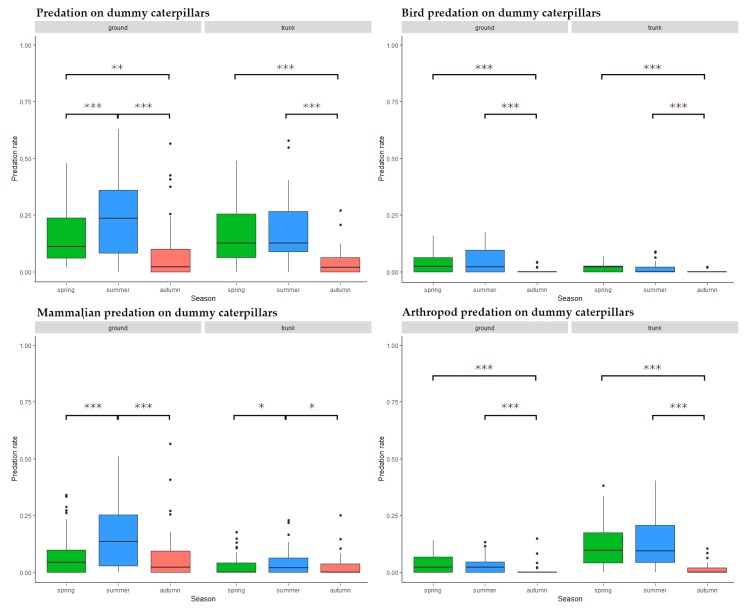
Predation activity during the season on different placement and by different predator groups. Significance codes: ***: *p* < 0.001, **: *p* < 0.01, *: *p* < 0.05, **˙**: *p* < 0.1.

Ground level predators were most active during summer (spring–summer: Estimate = 0.087, SD = 0.017, z = 5.078, *p* < 0.001, spring–autumn: Estimate = −0.057, SD = 0.019, z = −3.105, *p* = 0.005, summer–autumn: Estimate = −0.144, SD = 0.018, z = −7.881, *p* < 0.001) while on tree trunks, there was significantly lower activity during autumn (spring–summer: Estimate = 0.018, SD = 0.016, z = 1.151, p = 0.482, spring–autumn: Estimate = −0.117, SD = 0.017, z = −6.918, *p* < 0.001, summer–autumn: Estimate = −0.135, SD = 0.017, z = −7.824, *p* < 0.001) (Figure 5).

#### 3.2.2. Bird Predation on Dummy Caterpillars

Autumn predation levels were significantly lower than during spring or summer at both trunk (spring–summer: Estimate = −0.006, SD = 0.004, z = −1.620, *p* = 0.237, spring–autumn: Estimate = −0.020, SD = 0.004, z = −5.688, *p* < 0.001, summer–autumn: Estimate = −0.014, SD = 0.004, z = −3.942, *p* < 0.001) and ground level (spring–summer: Estimate = 0.006, SD = 0.008, z = 0.823, *p* = 0.689, spring–autumn: Estimate = −0.039, SD = 0.008, z = −5.007, *p* < 0.001, summer–autumn: Estimate = −0.045, SD = 0.008, z = −5.649, *p* < 0.001) (Figure 5).

#### 3.2.3. Mammalian Predation on Dummy Caterpillars

Mammal predation activity was highest during the summer months both at ground level (spring–summer: Estimate = 0.087, SD = 0.014, z = 6.018, *p* < 0.001, spring–autumn: Estimate < 0.001, SD = 0.015, z = 0.025, *p* = 1, summer–autumn: Estimate = −0.086, SD = 0.015, z = −5.786, *p* < 0.001) and on the trunks (spring–summer: Estimate = 0.018, SD = 0.007, z = 2.669, *p* = 0.021, spring–autumn: Estimate < −0.001, SD = 0.007, z = −0.038, *p* = 0.999, summer–autumn: Estimate = −0.018, SD = 0.007, z = −2.619, *p* = 0.024) (Figure 5). After closer examination the peak predation was between August and September, but the autumn decrease was steeper, as in the case of birds.

#### 3.2.4. Arthropod Predation on Dummy Caterpillars

Arthropod predators were less active during autumn than at other seasons, both at ground level (spring–summer: Estimate = 0.002, SD = 0.006, z = 0.293, *p* = 0.954, spring–autumn: Estimate = −0.025, SD = 0.006, z = −4.155, *p* < 0.001, summer–autumn: Estimate = −0.027, SD = 0.006, z = −4.310, *p* < 0.001) and on the trunks (spring–summer: Estimate = 0.005, SD = 0.014, z = 0.341, *p* = 0.938, spring–autumn: Estimate = −0.103, SD = 0.015, z = −6.745, *p* < 0.001, summer–autumn: Estimate = −0.108, SD = 0.016, z = −6.880, *p* < 0.001) (Figure 5).

## 4. Discussion

We found a generally decreasing predation activity along our urbanization gradient from rural to urban habitats except for ground-active arthropod predators (H2). This conforms to other findings [28,29] and does not indicate that communities regularly exposed to extreme flood events were insensitive to urbanization (H1). Urbanization can be a fast process, proceeding at a higher speed than natural landscape and environmental changes, creating highly dynamic and complex habitats [69]. The increasing disturbance hypothesis was supported by our data, except for arthropods at ground level, where Connell’s intermediate disturbance hypothesis [70] seemed a more acceptable explanation, predicting the increase in predation pressure at intermediate levels of disturbance, in the studied situation at the suburban sites. Alternatively, the “predation paradox” hypothesis [28,71] can also explain the decreasing predation pressure toward cities, but we have no information on the abundances of the different predator groups.

Ground beetles constitute one of the main arthropod predator group on the soil surface, together with spiders and ants [72,73]. We found traces of attack by ground beetles in this experiment. The majority of previous studies considering ground beetle abundance do not support the intermediate disturbance hypothesis [13,74,75]. Thus, there can be differences in movement activity (reflected by pitfall trap catches) and foraging activity (attacks on sentinel prey) in this group. Nevertheless, in the same study area, spiders also show a similar pattern which can be explained by Connell’s intermediate disturbance hypothesis [76] and ground beetle abundance was highest in rural habitats, moderate in suburban areas, and lowest in urban ones (S. Mizser, University of Debrecen, Debrecen, Hungary. Personal Communication, 2020.).

We found higher predation rates by birds in spring and summer than autumn. This result conformed to the match/mismatch hypothesis [51] (H3), except for the high predation rate during summer. This can be the result of the higher population densities just after the fledging of the nestlings. Slight differences in the date of laying the first egg (one week earlier in urban than rural habitats) and of the occurrence of peak caterpillar biomass (4 days later in urban habitats) [77] can slightly shift the results. For mammals, late summer was the most active period (H4). So, the population growth was not enough to increase the predation rate, but the increased protein needs during the reproduction period [78] can alter the eating habits of small mammals [79].

We found higher predation rates (14.6% overall, and 16.0% at ground level), than the previously reported 8.8% median predation rate on artificial prey [42]. This median was calculated from worldwide data and we have to consider that from the equator to the poles the predation activity in forested habitats gradually, but not significantly increases [42], so our result is not an outlier. Overall, we found higher predation rates at ground level which is also in agreement with previous results [42]. Vertebrate predation on artificial caterpillars (birds and mammals, 9.0% combined) was higher than arthropod predation (5.7%) as expected [42]. The same pattern was found at ground level (vertebrate predation 13.8%, arthropod predation 2.6%) but the opposite on tree trunk (vertebrate predation 4.2%, arthropod predation 8.8%). The higher arthropod predation above ground level is different from previously reported results [42]. This may reflect that higher small mammal activity at ground level probably included species that also prey on carabids and other ground-active arthropods, and their densities often show a negative relationship [80].

The urban predator assemblage is different from the natural and semi-natural communities [31]. Predator abundances and feeding habits are changing. For example, availability of anthropogenic food could, in the case of opportunistic predators, result in lower predation rates on certain taxa [81]. Other behavioural change can be triggered by more aggregated prey in urban habitats, for example around bird feeders [35] and light sources [34], which can result locally higher predation rates, and consequently lower predation activity at other places [37].

A rarely mentioned complication was that some caterpillars melted and had to be discarded—the soft plasticine cannot keep eventual attack marks. Initially, we attributed this to high temperatures—during sunny summer days, the temperatures at the study site can reach >35 °C. However, this effect may have been due to exposure to direct sunshine rather than high temperatures, because the same plasticine was successfully used in the Negev Desert in Israel, also at temperatures well above 40 °C (M. Ferrante, University of the Azores, Terceira, Portugal. Personal Communication, 2020).

In this riverside forested landscape, we found the same pattern, a decreasing predation pressure from rural to urban habitats, as earlier studies in non-riverside urbanized areas on birds as prey [28], but it is not clear that the changes in the riparian community composition were the same as in the other forest communities in other cities. Thus, it can be useful to compare riparian and non-riparian habitats in the same city in order to possibly separate these confounding effects. It would be profitable to investigate different prey groups, not only insects, but also amphibians, reptiles, mammals, and birds at the same time and place.

## 5. Conclusions

In conclusion, we found a decreasing predation pressure on artificial insect prey along the urbanisation gradient from rural to urban forest, with different predators being the most active at ground level vs. tree trunk. Our general attack rates tended to be higher than the median values in the literature. Our studied gradient was special in that it was in a floodbed crossing a large city. Apparently, the floods did not constitute a strong enough habitat filtering effect to overwrite the impact of habitat changes brought by urbanisation.

## Figures and Tables

**Table 1:** Tree and shrub species presence and the number of dummy caterpillars placed per session at the observed habitats on tree trunks. R—rural, S—suburban, U—urban, Y—given species is present at the location.

Common Name	Scientific Name	Number of Dummy Caterpillars On Tree Species in						Presence in Undergrowth in					
		R		S		U		R		S		U	
		1	2	1	2	1	2	1	2	1	2	1	2
Green ash *	*Fraxinus pennsylvanica*	24	1	30	16	7	4		Y		Y	Y	
Grey poplar	*Populus x canescens*	8	11	2	1	37	5				Y	Y	
White willow	*Salix alba*	15	3	8	6		32						
Box elder *	*Acer negundo*		25	7	7	2	4			Y	Y		Y
European White elm	*Ulmus laevis*	1	6	1	1	1							
Black poplar	*Populus nigra*		1		5	1							
White mulberry	*Morus alba*		1		3					Y			
Silver maple	*Acer saccharinum*				9		3				Y		
Field maple	*Acer campestre*										Y		
False indigo *	*Amorpha fruticosa*							Y	Y	Y	Y	Y	Y
Riverbank grape *	*Vitis riparia*									Y		Y	

* Invasive in Hungary [57].

**Table 2:** Predation pressure by different predator groups

	Predation Pressure (% prey Attacked) on Ground vs. Trunk		
Group	Overall (n = 12672)	Ground (n = 6336)	Trunk (n = 6336)
All predators	14.6	16.0	13.2
Birds	2.3	3.4	1.2
Mammals	6.7	10.4	3.0
Arthropods	5.7	2.6	8.8
Missing	3.5	5.4	1.6

## Appendix A

**Table A1:** Flooding history of river Tisza at Szeged between 1977–2016

Period	Floodbed Inundated (occasions)	Average Length of flood (days)	Inundation ≥50 cm (occasions)	Average Length of ≥50 cm Inundation (days)	Average Max Depth of Inundation (cm)
Whole year	25	44.6 (range 1–95)	20	41.0 (range 9–78)	173.1 (range 5–449)
April–October	24	35.6 (range 4–77)	20	31.9 (range 6–73)	172.1 (range 23–449)
